# Different roles of circulating and intramuscular GDF15 as markers of skeletal muscle health

**DOI:** 10.3389/fendo.2024.1404047

**Published:** 2024-05-14

**Authors:** Antonio Chiariello, Giuseppe Conte, Luca Rossetti, Lorenzo Trofarello, Stefano Salvioli, Maria Conte

**Affiliations:** ^1^ Department of Medical and Surgical Sciences (DIMEC), University of Bologna, Bologna, Italy; ^2^ Department of Agriculture, Food and Environment, University of Pisa, Pisa, Italy; ^3^ Interdepartmental Centre “Alma Mater Research Institute on Global Challenges and Climate Change (Alma Climate)”, University of Bologna, Bologna, Italy; ^4^ IRCCS Azienda Ospedaliero-Universitaria di Bologna, Bologna, Italy

**Keywords:** GDF15, biomarkers, muscle health, aging, inflammaging

## Abstract

**Introduction:**

Growth Differentiation Factor 15 (GDF15) is a mitokine expressed in response to various stresses whose circulating levels increase with age and are associated with numerous pathological conditions, including muscle wasting and sarcopenia. However, the use of circulating GDF15 (c-GDF15) as a biomarker of sarcopenia is still debated. Moreover, the role of GDF15 intracellular precursor, pro-GDF15, in human skeletal muscle (SM-GDF15) is not totally understood. In order to clarify these points, the association of both forms of GDF15 with parameters of muscle strength, body composition, metabolism and inflammation was investigated.

**Methods:**

the levels of c-GDF15 and SM-GDF15 were evaluated in plasma and muscle biopsies, respectively, of healthy subjects (HS) and patients with lower limb mobility impairment (LLMI), either young (<40 years-old) or old (>70 years-old). Other parameters included in the analysis were Isometric Quadriceps Strength (IQS), BMI, lean and fat mass percentage, *Vastus lateralis* thickness, as well as circulating levels of Adiponectin, Leptin, Resistin, IGF-1, Insulin, IL6, IL15 and c-PLIN2. Principal Component Analysis (PCA), Canonical Discriminant Analysis (CDA) and Receiving Operating Characteristics (ROC) analysis were performed.

**Results:**

c-GDF15 but not SM-GDF15 levels resulted associated with decreased IQS and IGF-1 levels in both HS and LLMI, while only in LLMI associated with increased levels of Resistin. Moreover, in LLMI both c-GDF15 and SM-GDF15 levels were associated with IL-6 levels, but interestingly SM-GDF15 is lower in LLMI with respect to HS. Furthermore, a discrimination of the four groups of subjects based on these parameters was possible with PCA and CDA. In particular HS, LLMI over 70 years or under 40 years of age were discriminated based on SM-GDF15, c-GDF15 and Insulin levels, respectively.

**Conclusion:**

our data support the idea that c-GDF15 level could be used as a biomarker of decreased muscle mass and strength. Moreover, it is suggested that c-GDF15 has a different diagnostic significance with respect to SM-GDF15, which is likely linked to a healthy and active state.

## Introduction

Aging is a complex process characterized by multiple biological alterations, including changes in body composition. In particular, during aging, a decrease of muscle mass and strength and an increase of total fat mass occur (1–3). Over time, these changes may contribute to the onset of different age-related diseases, and in particular sarcopenia (4). The causes of sarcopenia are not yet completely understood, but to date, it is thought to be a multifactorial disease, in which neurological decline, hormone changes, increased inflammatory status, decline in physical activity and poor nutrition, together contribute to its onset (5). Sarcopenia is becoming highly prevalent in the elderly population, with a remarkable impact on the quality of life, since it is associated with increase of falls, poor physical function and difficulties in activities of daily living. Moreover, it is considered as both a precursor and a physical manifestation of frailty, and its diagnostic process is often difficult (6, 7). Given the impact of this pathology, in particular on Western societies that are rapidly aging, and the fact that elderly population is continuously increasing, prevention, early diagnosis and treatment of sarcopenia are becoming fundamental.

A chronic and low-grade state of inflammation, known as “inflammaging” (8), is considered one of the hallmarks of aging and one of the main drivers of many, if not all, age-related diseases, including sarcopenia. In fact, elevated circulating levels of typical inflammaging mediators, such as Interleukin 6 (IL6), Tumor necrosis factor α (TNFα) and C-reactive protein (CRP), have been found in patients with sarcopenia (9–11). However, inflammaging has also a counterpart indicated as anti-inflammaging, whose components are still under investigation, though some of them have been possibly identified, such as Growth Differentiation Factor 15 (GDF15) (12, 13). Recently, a possible association of GDF15 with sarcopenia and muscle mass loss has been reported (14–16). GDF15 is a mitokine, *i.e.* a stress-related protein mainly secreted in response to mitochondrial stress, and one of the most upregulated proteins during aging and is associated with overall mortality in the elderly (17–19). Human *GDF15* gene encodes for a 308 amino acid biologically inactive precursor protein, named pre-pro-GDF15, which is cleaved in the endoplasmic reticulum to pro-GDF15. At the C-terminal a conserved domain of seven consecutive cysteines makes the pro-GDF15 to form an intracellular homodimer (35 kDa) through a single disulfide bond (20). The pro-GDF15 is further processed in the trans-Golgi apparatus and secreted as mature GDF15 homodimer (15 kDa) (21, 22). As mentioned, a tight relationship of GDF15 with inflammation has been found, since it has been shown that its levels increase in response to infections and inflammation, with possible immunoregulatory and immunosuppressive roles, being fundamental in tolerance and adaptation to bacterial and viral infections (23, 24). Other precise biological functions of GDF15 are still poorly defined, but it has been shown that it acts centrally to control appetite and is involved in energy metabolism, with catabolic and cachexia-promoting effects (13, 25). Many studies have associated GDF15 with a plethora of age-related diseases, such as neurodegenerative diseases, cardiovascular diseases, metabolic diseases and cancer (13, 26–29).

As mentioned, an association of the circulating GDF15 (c-GDF15), *i.e.* the mature GDF15 homodimer, with sarcopenia and muscle atrophy has been suggested. In particular, studies showed that c-GDF15 level could easily predict sarcopenia in patients with chronic obstructive pulmonary disease (30) and in aged mice and humans (31). Moreover, inactivation of GDF15 signaling in a mouse model of cancer-induced cachexia led to improved muscle mass and physical performance (16). However, the usefulness of c-GDF15 as a biomarker of sarcopenia is still debated, as it is not possible to understand which tissue it comes from. In fact, GDF15, as pro-GDF15 form, is expressed by many tissues, including skeletal and cardiac muscles, and consequently it is listed among myokines (32). Moreover, it is not clear what is the role, if any, of intracellular pro-GDF15 protein in human skeletal muscle (SM-GDF15). In particular, it is not clear whether SM-GDF15 expression is just a sign of muscle stress and reflects c-GDF15 levels, or rather it may play a detrimental or beneficial role trying to promote or counteract muscle wasting and weakness. To this aim, in the present study we have analyzed the levels of c-GDF15 and SM-GDF15 in both healthy subjects with an active life-style and patients with lower limb mobility impairment and a sedentary life-style, with different age (from 20 to 96 years). We have found that in patients c-GDF15 level is higher while, surprisingly, SM-GDF15 level is lower compared to healthy subjects. Moreover, c-GDF15 level is inversely correlated with muscle strength in both healthy subjects and patients, supporting its use as a biomarker of sarcopenia and muscle dysfunction.

## Methods

### Subjects’ description

The subjects were recruited in the framework of the EU Project “MYOAGE”. The study protocol was approved by the Ethical Committee of Istituto Ortopedico Rizzoli, Bologna, Italy (ethical clearance no. 10823 issued on April 26, 2010). All subjects signed an informed consent before entering the study. For this study, ethical aspects for aging research were considered, as illustrated in 33.

Samples from two groups of subjects were used: i) 47 healthy subjects (HS) (mean age 57.1 ± 3.6) divided in 15 subjects <40 years of age (mean age 22.7 ± 0.63) and 32 subjects >70 years of age (mean age 74.3 ± 0.62); ii) 46 patients with lower limb mobility impairment (LLMI) (mean age 61.3 ± 3.6), divided in 21 subjects <40 years of age (mean age 36.3 ± 1.5) and 25 subjects >70 years of age (mean age 82.2 ± 1.6). To ensure the selection of subjects in healthy conditions, the following exclusion criteria were used: presence of comorbidities as detailed in 2, inability to walk a distance of 250m, use of medication, immobilization for 1 week during the last 3 months and orthopedic surgery during the last 2 years. For the patient’s group, exclusion criteria were: the presence of chronic kidney or liver diseases, bleeding disorders, severe diabetes mellitus, rheumatic diseases other than osteoarthritis, neuromuscular disorders, malignancies and systemic infections, chronic steroid use, major psychological problems or history of alcohol or drug abuse, evidence of prior surgery in the involved hip (2).

Height and weight were measured for each subject and BMI was calculated as weight in kilograms divided by the square of the height in meters (kg/m2).

### Laboratory measurements on plasma

Blood samples were collected in the morning after an overnight fasting. Plasma samples were obtained after a 15 minutes centrifugation at 2,000 g at 4°C, then rapidly frozen and stored at -80°C until the analysis was performed. Plasma IGF-1, Insulin, Adiponectin, Leptin, Resistin, IL6 and GDF15 concentrations were obtained using commercial ELISA kits (Quantikine R&D Systems), according to manufacturer’s instructions. Circulating Perilipin 2 (c-PLIN2) was measured using the ELISA commercial kit Human ADRP ELISA (E-EL-H0278, Elabscience), according to the manufacturer’s instructions. Each analyte was measured in duplicate for each sample. Plasma IL15 was analyzed using the Simple Plex Human IL-15 Cartridge (ProteinSimple/Bio-Techne) run on an Ella Automated Immunoassay System (ProteinSimple/Bio-Techne), according to manufacturer’s instructions.

### Muscle strength

For HS, isometric quadriceps strength (IQS) was measured with a quadriceps chair (Forcelink B.V). Briefly, the subjects were positioned in an upright position, with straps to fix the hips to the chair and the ankle to the force transducer, at the knee angle of 90 degrees. Three trials were conducted to measure maximal voluntary contraction of the quadriceps. Each trial was separated by one minute of rest. The trial with the highest force output was taken for analyses.

For LLMI, the IQS was measured in seated position using a Handifor dynamometer (TRACTEL S.A. Montreuil Cedex – France). After a warming up period, patients were asked to perform three series of 10 contractions, progressively increasing the strength developed. The highest peak torque was withheld for analyses.

### Muscle ultrasound measurements

Only for LLMI, ultrasound imaging of the *Vastus lateralis* (VL) was performed using a portable ultrasound (Mylab25, Esaote) with a 7–10 MHz linear probe. Acquisition was performed by a trained examiner. Muscle thickness was calculated as the vertical distance between muscle superficial and deep aponeuroses, at an equidistant point from right and left borders of the sagittal image.

### Dual-energy x-ray absorptiometry

Only for HS, a whole-body scan to detect total fat and lean mass was performed. The scan was performed using Dual-energy X-ray absorptiometry (DXA) (Hologic QDR 4500, version 12.4, Hologic Inc., Bedford), by a trained technician.

### Muscle biopsies sampling

Muscle biopsies were taken from VL muscle from 23 HS, after localized anesthesia, and from 16 LLMI, during the operation at the site of surgical incision. All biopsies were immediately frozen in liquid nitrogen and then stored at -80°C.

### Protein extraction and western blotting

Proteins were obtained by lysis of about 40mg of frozen tissue using TEAD buffer (Tris-HCl 20 mM pH= 7.5, EDTA 1mM, NaN3 1mM, DTT 1mM) with protease and phosphatase inhibitors (Sigma). Homogenization was performed using a motor driven homogenizer and lysates obtained were then centrifuged at 25,000 g for 1h at 4°C.

10 μg of total proteins were separated on a polyacrylamide gel (4-15% Mini-PROTEAN^®^ TGX™ Precast Protein Gels, Bio-Rad). Proteins were then transferred to a nitrocellulose membrane (Trans-Blot Transfer Medium, Bio-Rad) and then immunoblotted with anti-Mic-1/GDF15 (Cell Signaling) primary antibody. Anti-GAPDH primary antibody (Novus Bio) was used as housekeeping for normalization.

Images acquisition was performed with ChemiDoc Imaging System (Bio-Rad). Band densitometry analysis was performed with Fiji software.

### Statistical analysis

Results are shown as mean ± SD or SE. After a Shapiro-Wilk normality test, Student’s t test, and Pearson correlation were used for normally distributed data whereas Mann-Whitney test was used for data that did not follow a normal distribution. SPSS 17.0 for Windows was used for analyses. P values <0.05 were considered statistically significant. The *post-hoc* power calculation, performed with G*Power software, was 0.81, considering an effect size of 0.30.

To understand the role of c-GDF15 and SM-GDF15 in relation to other parameters (Insulin, Leptin, c-PLIN2, IL6, IL15, Resistin, Adiponectin and BMI) involved in muscle strength and mass loss, a principal component analysis (PCA) was used. The PCA is a multivariate dimension reduction application, mainly aimed at synthesizing data contained in a set of n observed variables (y1,…, yn) by checking a new set of p (p < n) variables (X1,…,Xp), named principal components (PCs). The first PC (PC1) explains the highest variability, while the remaining PCs (PC2, PC3,… PCn; n = number of variables) report for the remaining variability in the data. Each PC is independent and orthogonal to the others. Generally, the first few PCs are sufficient to describe most of the total data variations (34).

Subsequently, a Canonical Discriminant Analysis (CDA) was used to evaluate which of the variables involved in the PCA were able to best discriminate the 4 groups of subjects involved in the test (healthy young subjects, young patients, healthy old subjects and old patients). The CDA is a dimension-reduction technique, related to principal component analysis and canonical correlation, able to perform both univariate and multivariate 1-way analyses. Given a classification character and several interval variables, CDA derives a set of new variables, called canonical functions (CAN), which are linear combinations of the original interval variables.

Moreover, receiver operating characteristics (ROC) curves were constructed to assess the cut-off levels and the discriminatory ability of the above-mentioned parameters in HS and LLMI.

## Results

### c-GDF15 levels and SM-GDF15 protein follow different trends in patients and healthy subjects

We have previously reported that the level of c-GDF15 in patients with lower limb mobility impairment (LLMI) is higher than that of healthy subjects (HS), and that in both LLMI and HS, older subjects have higher levels of c-GDF15 with respect to younger ones (14). The analysis of c-GDF15 in the present study confirmed previous results, *i.e.* higher c-GDF15 levels in LLMI *vs* HS and in >70yrs *vs <*40yrs subjects (**Supplementary Figures 1A–C**).

**Figure 1:**
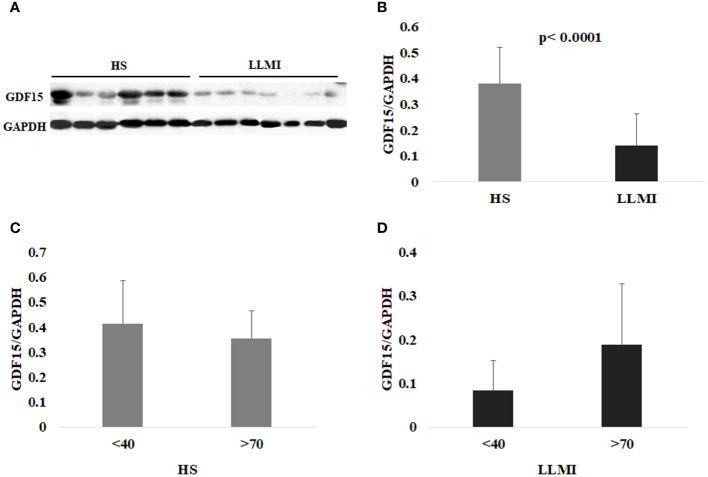
Western blotting analysis of GDF15 in the skeletal muscle (SM-GDF15). **(A)** Representative immunoblotting image of GDF15 and GAPDH in the skeletal muscle. **(B)** Relative protein expression of GDF15 in the SM from 23 healthy subjects (HS) and 16 patients with lower limb mobility impairment (LLMI). **(C)** Relative protein expression of GDF15 in the SM from 10 HS <40 years (<40) and 13 HS >70 years (>70). **(D)** Relative protein expression of GDF15 in the SM from 7 LLMI <40 and 9 LLMI >70. The bars represent mean ± SD. The quantification was performed using Fiji software and normalized to GAPDH expression. Student’s t test was applied.

We then wondered whether the same trend was also present in skeletal muscle tissue. To this purpose, we evaluated, in the same subjects, the protein level of the intracellular pro-GDF15 form within skeletal muscle (SM-GDF15), analyzing the whole protein extract from VL biopsies by western blotting. Surprisingly, the level of SM-GDF15 followed a different trend compared to c-GDF15. In particular, LLMI showed a significantly lower level of SM-GDF15 as compared to HS (**Figures 1A, B**). Moreover, no significant differences were found between younger (<40 yrs) and older (>70yrs) subjects in both HS and LLMI groups (**Figures 1C, D**). These results indicate that actually GDF15 protein follows different concentration trends in plasma and skeletal muscle. Moreover, no correlation between SM-GDF15 and c-GDF15 was observed (see below).

### c-GDF15 correlates with markers of inflammation and muscle functionality

We then sought for correlations of c-GDF15 and SM-GDF15 with biochemical, anthropometric and functional parameters [age, BMI, Isometric Quadriceps Strength (IQS), Adiponectin, Leptin, Resistin, Insulin, IL6, IL15, c-PLIN2, IGF-1] related to inflammation, metabolism, body composition and muscle functionality. Mean values of the above-mentioned parameters, analyzed in HS and LLMI, are summarized in **Table 1**.

**Table 1:** Summary of biochemical and anthropometric parameters of HS and LLMI patients. Data are expressed ad mean ± SE.

	HS (47 subjects)	LLMI (46 subjects)
**Age (years)**	57.1 ± 3.6	61.3 ± 3.6
**BMI (kg/m^2^)**	24.9 ± 0.6	26.0 ± 0.6
**c-GDF15 (pg/ml)**	1168.1 ± 87.6	2021.6 ± 249.1
**IQS (kg)**	146.5 ± 10.4	26.3 ± 1.7
**Adiponectin (µg/ml)**	13.7 ± 1.2	10.7 ± 1
**Leptin (pg/ml)**	11.7 ± 1.9	12.2 ± 1.4
**Resistin (ng/ml)**	7.1 ± 0.4	9.3 ± 1.1
**IGF-1 (ng/ml)**	122.5 ± 9.7	117.5 ± 8.4
**Insulin (µU/ml)**	2.1 ± 0.03	12.6 ± 2.1
**IL6 (pg/ml)**	3.2 ± 0.2	16.7 ± 3.7
**IL15 (pg/ml)**	2.0 ± 0.1	4.0 ± 1.8
**c-PLIN2 (ng/ml)**	32.2 ± 4.9	44.7 ± 5.3

**Figure 2:**
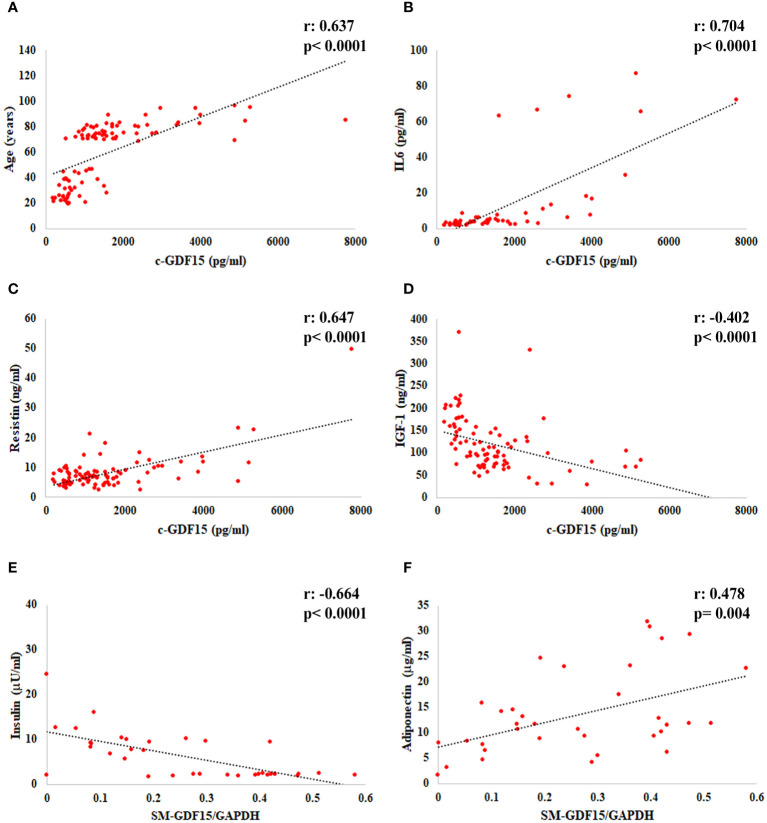
Linear regression analysis of c-GDF15 with **(A)** age, **(B)** IL6, **(C)** Resistin, **(D)** IGF-1, and of SM-GDF15 with **(E)** Insulin and **(F)** Adiponectin, in HS and LLMI. Pearson correlation coefficient (r) and p-value are shown.

When we considered all the subjects analyzed (LLMI+HS), a positive correlation of c-GDF15 with age, IL6 and Resistin and a negative correlation with IGF-1 were observed. As far as SM-GDF15, a positive correlation with Adiponectin and a negative correlation with Insulin were observed (**Figures 2A–F**).

When we considered HS group only, a positive correlation of c-GDF15 with age, BMI and IL6 and a negative one with IQS, IQS/BMI and IGF-1 were observed. For this group, data on body composition were available. Interestingly, SM-GDF15 correlated with fat percentage and inversely with lean percentage (27.6% ± 1.3 and 70.4% ± 1.3 of HS body composition on average, respectively) (**Table 2**).

**Table 2:** Pearson correlations of c-GDF15 and SM-GDF15 with the metabolic and inflammatory parameters in HS (r: Pearson correlation coefficient; p: p-value).

	c-GDF15		SM-GDF15	
	r	p	r	p
**Age**	**0.756**	**< 0.0001**	-0.083	0.744
**BMI**	**0.300**	**0.041**	-0.196	0.436
**IQS (kg)**	**-0.325**	**0.026**	-0.426	0.078
**IQS/BMI**	**-0.381**	**0.008**	-0.342	0.165
**Fat %**	0.165	0.272	**0.517**	**0.028**
**Lean %**	-0.118	0.435	**-0.527**	**0.025**
**Adiponectin**	0.122	0.413	0.322	0.192
**Leptin**	0.154	0.301	0.389	0.110
**Resistin**	0.131	0.379	-0.005	0.984
**IGF-1**	**-0.594**	**< 0.0001**	0.059	0.818
**Insulin**	-0.093	0.534	0.334	0.176
**c-PLIN2**	0.152	0.330	0.299	0.243
**IL6**	**0.640**	**< 0.0001**	0.123	0.639
**IL15**	-0.055	0.104	0.104	0.896
**SM-GDF15**	-0.199	0.444	–	–

Statistically significant values are indicated in bold.

When we considered LLMI group only, a positive correlation of c-GDF15 with age, Resistin and IL6 and a negative one with IQS, IQS/BMI and IGF-1 were observed. For this group, data on VL thickness were available (1.3mm ± 0.1 on average). An inverse correlation of c-GDF15 with this parameter was found. Furthermore, a positive correlation between SM-GDF15 and IL6 was found (**Table 3**). Interestingly, higher IL6 plasma levels were observed in LLMI with respect to HS (p= 0.00095, Student’s t test. **Supplementary Figure 2**).

**Table 3:** Pearson correlations of c-GDF15 and SM-GDF15 with the metabolic and inflammatory parameters in LLMI (r: Pearson correlation coefficient; p: p-value).

	c-GDF15		SM-GDF15	
	r	p	r	p
**Age**	**0.714**	**< 0.0001**	0.378	0.135
**BMI**	-0.263	0.081	0.106	0.685
**IQS (kg)**	**-0.405**	**0.019**	-0.188	0.628
**IQS/BMI**	**-0.469**	**0.006**	-0.164	0.673
**Muscle thickness**	**-0.551**	**< 0.0001**	-0.028	0.921
**Adiponectin**	0.192	0.213	0.284	0.308
**Leptin**	-0.076	0.623	0.263	0.308
**Resistin**	**0.689**	**< 0.0001**	0.086	0.743
**IGF-1**	**-0.403**	**0.013**	-0.205	0.482
**Insulin**	-0.144	0.350	-0.421	0.092
**c-PLIN2**	-0.019	0.908	0.291	0.292
**IL6**	**0.687**	**< 0.0001**	**0.503**	**0.039**
**IL15**	-0.105	0.493	-0.336	0.188
**SM-GDF15**	0.366	0.148	–	–

Statistically significant values are indicated in bold.

No correlation was observed with either c-PLIN2 (a marker of adiposity according to 28) or IL15 (a positive modulator of muscle growth, according to 35). Taken together, these data indicate that c-GDF15 is correlated with decreased muscle strength and increased inflammation and could thus represent a biomarker of poor muscle function.

### Principal component and canonical discriminant analyses separate HS and LLMI

To better understand the diagnostic role and the possible link of c-GDF15 and SM-GDF15 with the other parameters analyzed, we performed a principal component analysis (PCA) and a canonical discriminant analysis (CDA).

**Figure 3:**
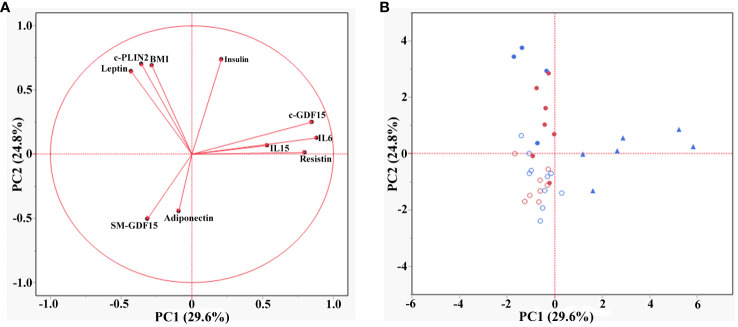
Principal component analysis (PCA) in HS and LLMI. **(A)** Loading plot of the PCA showing the different groups of parameters. **(B)** Score plot of the PCA showing the association of the different groups of subjects (<40 years HS, >70 years HS, <40 years LLMI and >70 years LLMI) with the parameters analyzed. Empty red circles are HS <40 years; empty blue circles are HS >70 years; filled red circles are LLMI <40 years; filled blue circles are LLMI >70 years; filled triangles are LLMI >85 years.

PCA was applied to explore the effect of the parameters analyzed in both HS and LLMI subjects. The first two principal components (PC1 and PC2) described 54% of the total variation (29.6% and 24.8% for PC1 and PC2 respectively). The loading plot of the PCA showed that the parameters analyzed could be grouped into three main groups (**Figure 3A**). The first group (positive loadings for PC1 and PC2) included c-GDF15, IL6, IL15 and Resistin, which are molecules related to inflammatory responses (23, 36–38). The second group (variables with negative and positive loadings for PC1 and PC2 respectively) included BMI, c-PLIN2 and Leptin, that are related to adipose tissue and its metabolism, as well as energy balance regulation (39, 40). The third group (negative loadings for both PCs) included Adiponectin, an anti-inflammatory protein secreted by adipose tissue, and SM-GDF15. Surprisingly, the score plot of the PCA showed that HS were mainly associated with SM-GDF15 and Adiponectin, while LLMI were associated with all the other parameters. These latter could be further subdivided by age, in particular >70yrs LLMI were mainly associated to c-GDF15, IL6, IL15 and Resistin (**Figure 3B**).

**Figure 4:**
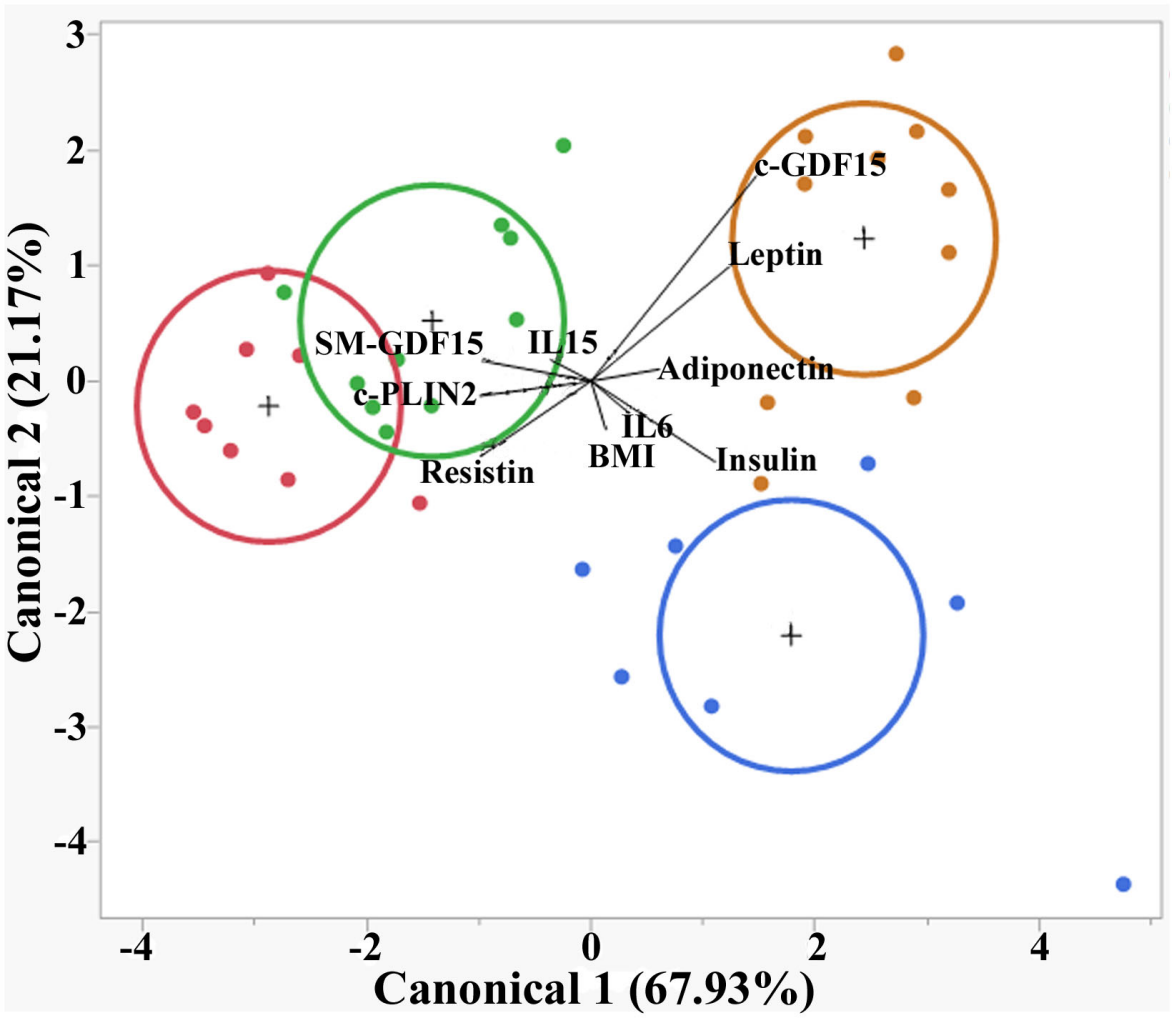
Canonical discriminant analysis (CDA) in HS and LLMI. Discrimination of <40 HS, >70 HS, <40 LLMI and >70 LLMI based on the parameters analyzed. Red circle: <40 HS; green circle: >70 HS; blue circle: <40 LLMI; yellow circle: >70 LLMI.

CDA showed that the parameters considered could discriminate the subjects into four groups (<40yrs HS, >70yrs HS, <40yrs LLMI and >70yrs LLMI). The canonical 1 accounted for 67.93% of separation and the canonical 2 for 21.17%. In particular, >70yrs LLMI were well separated from all other groups, mainly because of c-GDF15 and Leptin expression levels (**Figure 4**).

**Figure 5:**
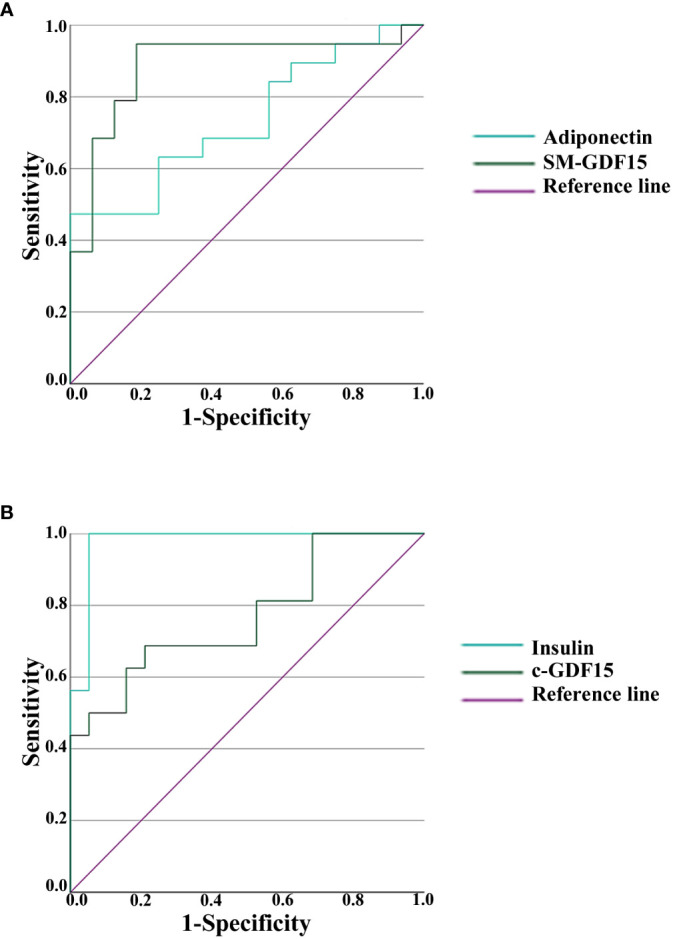
Receiver operating characteristic (ROC) analysis in HS and LLMI. **(A)** ROC curves of Adiponectin and SM-GDF15 in HS. **(B)** ROC curves of Insulin and c-GDF15 in LLMI.

In order to evaluate which of the parameters analyzed had the highest discriminative ability in HS and LLMI, we calculated the receiver operating characteristic (ROC) curves, also in order to detect a cut-off for the discriminating parameters considered. The areas under the curves (AUCs) are reported in **Tables 4**, **5**. SM-GDF15 and Adiponectin resulted the most discriminative parameters for HS (**Figure 5A**), while Insulin and c-GDF15 were those for LLMI (**Figure 5B**). These data indicate that these parameters can discriminate between LLMI and HS and, interestingly, that c-GDF15 and SM-GDF15 levels seem to characterize LLMI and HS, respectively.

**Table 4:** ROC analysis of metabolic and inflammatory parameters in HS.

Test result variables	Area (AUCs)	Std. Error	p
**Adiponectin**	**0.734**	0.084	**0.019**
**Leptin**	0.507	0.103	0.947
**Resistin**	0.480	0.106	0.843
**Insulin**	0.023	0.024	0.000
**c-GDF15**	0.230	0.082	0.007
**SM-GDF15**	**0.888**	0.062	**0.000**
**IL6**	0.219	0.089	0.005
**c-PLIN2**	0.438	0.100	0.529

Areas under the curves (AUCs) are shown. Null hypothesis: true area = 0.5.

Statistically significant values are indicated in bold.

**Table 5:** ROC analysis of metabolic and inflammatory parameters in LLMI patients.

Test result variables	Area (AUCs)	Std. Error	p
**Adiponectin**	0.266	0.084	0.019
**Leptin**	0.493	0.103	0.947
**Resistin**	0.520	0.106	0.843
**Insulin**	**0.977**	0.024	**0.000**
**c-GDF15**	**0.770**	0.082	**0.007**
**SM-GDF15**	0.112	0.062	0.000
**IL6**	0.781	0.089	0.005
**c-PLIN2**	0.562	0.100	0.529

Areas under the curves (AUCs) are shown. Null hypothesis: true area = 0.5. The test result variable IL6 has at least one tie between the positive actual state group and the negative actual state group, statistic may be biased.

Statistically significant values are indicated in bold.

## Discussion

It has been reported that plasma levels of GDF15 are associated with low muscle function and sarcopenia (14, 41–45). However, it is not yet clear what is the role of SM-GDF15 in muscle function and whether the levels of c-GDF15 are linked to SM-GDF15, given that skeletal muscles constitute the largest body component. Therefore, in this study we addressed the following questions: i. are the levels of SM-GDF15 correlated with those of c-GDF15? ii. are the levels of SM-GDF15 correlated with muscle strength or other parameters related to inflammation or metabolism? iii. is there any change with age in SM-GDF15 and its possible correlations with other parameters? iv. is it possible to discriminate patients suffering of muscle disuse or sarcopenia from healthy controls, as well as young from elderly subjects taking advantage of these parameters?

**Figure 6:**
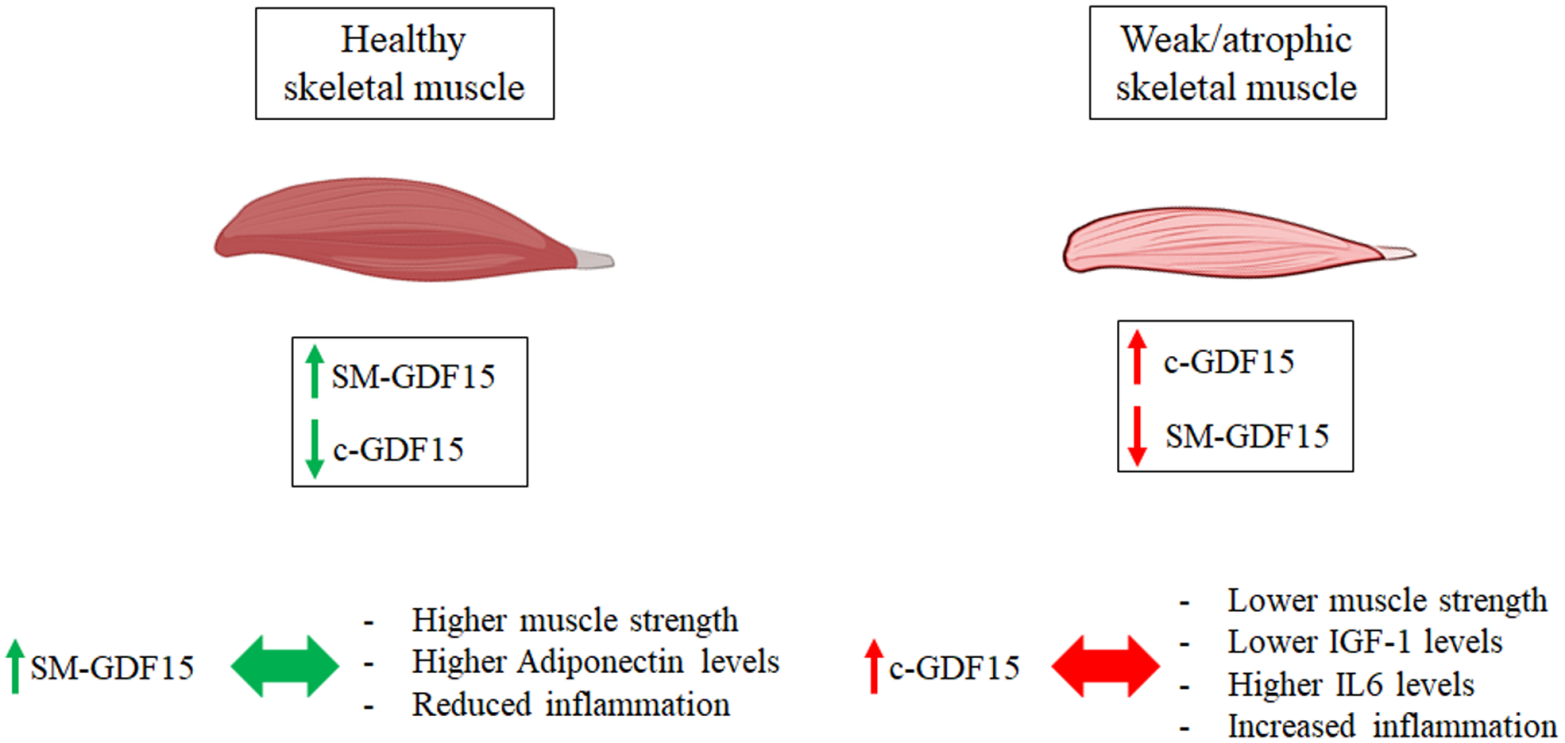
Working hypothesis on the diagnostic roles of c-GDF15 and SM-GDF15. Higher levels of SM-GDF15 and lower levels of c-GDF15 are observed in healthy subjects with functional muscles, and are associated with reduced inflammation, higher muscle strength and higher Adiponectin levels. Lower SM-GDF15 and higher c-GDF15 levels, on the contrary, are observed in patients with LLMI, with atrophic and weak muscles, and are associated with increased inflammation, higher IL6 levels and lower IGF-1 levels and muscle strength.

Quite surprisingly, SM-GDF15 expression evaluated in *Vastus lateralis* biopsies did not correlate with the levels of c-GDF15 nor with isometric quadriceps strength (IQS) and resulted higher in HS than LLMI samples. This suggests that the contribution of skeletal muscle to the production of c-GDF15 is likely exceeded by the contributions of other organs/tissues. Moreover, given the fact that the expression of SM-GDF15 is apparently higher in HS group, it is possible that in skeletal muscle such an expression is stimulated by fiber contraction, as suggested also by previous data indicating a transient spike of GDF15 expression upon a strenuous physical bout (14). On the contrary, it seems that inactivity induces a much lower expression of SM-GDF15. This conclusion is schematized in **Figure 6**.

As far as the correlation with other parameters, when considering LLMI and HS together, c-GDF15 is associated with age, as expected, and with other parameters such as IGF-1 (46), IL6 (47), Resistin (46), a finding that is also not unexpected, as these parameters are associated with age, too. At variance, SM-GDF15 resulted directly associated with Adiponectin and inversely with Insulin. In the case of Adiponectin, it is known that it plays a role in maintaining skeletal muscle health and function, in particular by promoting glucose uptake and fatty acids oxidation in the skeletal muscle (48, 49). Given that a role in promoting lipid oxidation has also been attributed to GDF15 (50), it is possible that the two molecules could have a synergic role in the skeletal muscle. As far as Insulin, it is known that GDF15 improves insulin sensitivity (51, 52), thus it is possible that an inverse correlation exists between Insulin and GDF15. However, it is to note that existing data regard c-GDF15, but not SM-GDF15, therefore this hypothesis has to be further validated. Interestingly, c-GDF15 is inversely associated with IQS in both HS and LLMI, while SM-GDF15 positively correlates with IL6 only in LLMI but not HS group. Together with the observation that SM-GDF15 seems to be higher in HS, these data may suggest that c-GDF15 but not SM-GDF15 is associated with inflammation and decreased muscle function (**Figure 6**). In particular, the fact that in LLMI, but not in HS group, SM-GDF15 is associated with IL6 may suggest that a normal physical activity induces GDF15 but not IL6 expression, while chronic inactivity induces IL6 but not (or not so much) GDF15. Unfortunately, we do not have data on muscle-specific expression of IL6, however, being IL6 a well-known myokine needed to induce muscle repair (53, 54), it can be inferred that, in absence of overt infections, the large majority of circulating IL6 comes from muscles. Since GDF15 expression is triggered, among others, by mitochondrial stress (55–57), it can be hypothesized that GDF15 expression is induced in contracting myofibers, where an intense mitochondrial activity is present. On the contrary, inactive muscle may display a reduced GDF15 expression but, on the contrary, a higher IL6 expression. Further experiments are needed to formally prove this hypothesis.

It is important to note that in skeletal muscle tissue we were only able to detect the immature and non-cleaved form of GDF15 (pro-GDF15) and not the mature form, which is the one found in the circulation and considered the most active. Thus, we cannot rule out the possibility that SM-GDF15 (being an immature form) is not proportionally connected with the levels of c-GDF15 (therefore accounting for the missing association between the two forms), however, it is known that many other organs and tissues produce this protein, including prostate, kidney, lung, but also senescent cells and many types of cancers (13, 26, 58–60). Therefore, the level of c-GDF15 derives from the sum of all these contributions, reflecting the general health state of a subject rather than muscle health alone.

To further support the idea that the diagnostic meaning of c-GDF15 and SM-GDF15 are different, the PCA, CDA and ROC analysis clearly indicate that these two variables have an opposite direction and can help discriminating between HS and LLMI groups. In particular, c-GDF15 is the main factor for discriminating >70yrs patients, while <40yrs patients seem to be discriminated by other parameters including Insulin and IL6. At variance, SM-GDF15 seems to be associated with muscle activity and can help discriminating healthy, active people.

As a whole, these data suggest that c-GDF15 is associated with decreased muscle strength and mass and can be useful to identify patients with muscle function impairment/sarcopenia, in particular elderly ones (**Figure 6**).

### Limitations of the study

This study has some limitations. First of all, the relatively low number of subjects that have been studied and the lack of measurement of some parameters, such as IL6 in skeletal muscle biopsies, due to the tiny amount of biopsy material available. However, the *post-hoc* power analysis indicated that the sample numerosity was high enough to grant for trustable results. The exact role of GDF15 within the muscle fibers remains poorly elucidated and the present data seem apparently in contrast with the well-known pro-cachectic and catabolic role of GDF15. It is however to note that the conclusions of the majority of studies stem from experiments only considering c-GDF15, not SM-GDF15. Whether the level of this intramuscular form of GDF15 has precise biologic/metabolic effects at muscle level or it is rather a mere marker of muscle activity needs to be better clarified with further studies.

## Data availability statement

The raw data supporting the conclusions of this article will be made available by the authors, without undue reservation.

## Ethics statement

The studies involving humans were approved by the Ethical Committee of Istituto Ortopedico Rizzoli, Bologna, Italy (ethical clearance no. 10823 issued on April 26, 2010). The studies were conducted in accordance with the local legislation and institutional requirements. The participants provided their written informed consent to participate in this study.

## Author contributions

AC: Data curation, Formal analysis, Investigation, Methodology, Writing – original draft. GC: Data curation, Formal analysis, Software, Writing – review & editing. LR: Investigation, Methodology, Writing – review & editing. LT: Investigation, Methodology, Writing – review & editing. SS: Conceptualization, Formal analysis, Funding acquisition, Supervision, Writing – original draft. MC: Conceptualization, Formal analysis, Supervision, Writing – original draft.

## Supplementary material

Supplementary Figure 1 ELISA analysis of c-GDF15 levels in healthy subjects (HS) and patients with lower limb mobility impairment (LLMI). **(A)** c-GDF15 level in LLMI compared to HS. **(B)** c-GDF15 level in HS of <40 years of age (<40) compared to HS of >70 years of age (>70). **(C)** c-GDF15 level in LLMI <40 compared to LLMI >70. Data are expressed as mean ± SD. Mann-Whitney test was applied. Each sample was analyzed in duplicate.

Supplementary Figure 2 ELISA analysis of plasma IL6 levels in HS and LLMI. Data are expressed as mean ± SD. Student’s t test was applied. Each sample was analyzed in duplicate.
