# Supplementary figures and images for: Different roles of circulating and intramuscular GDF15 as markers of skeletal muscle health
Source: Front Endocrinol (Lausanne). 2024 May 14;15:1404047. doi: 10.3389/fendo.2024.1404047 (PMC11130406; doi:10.3389/fendo.2024.1404047)

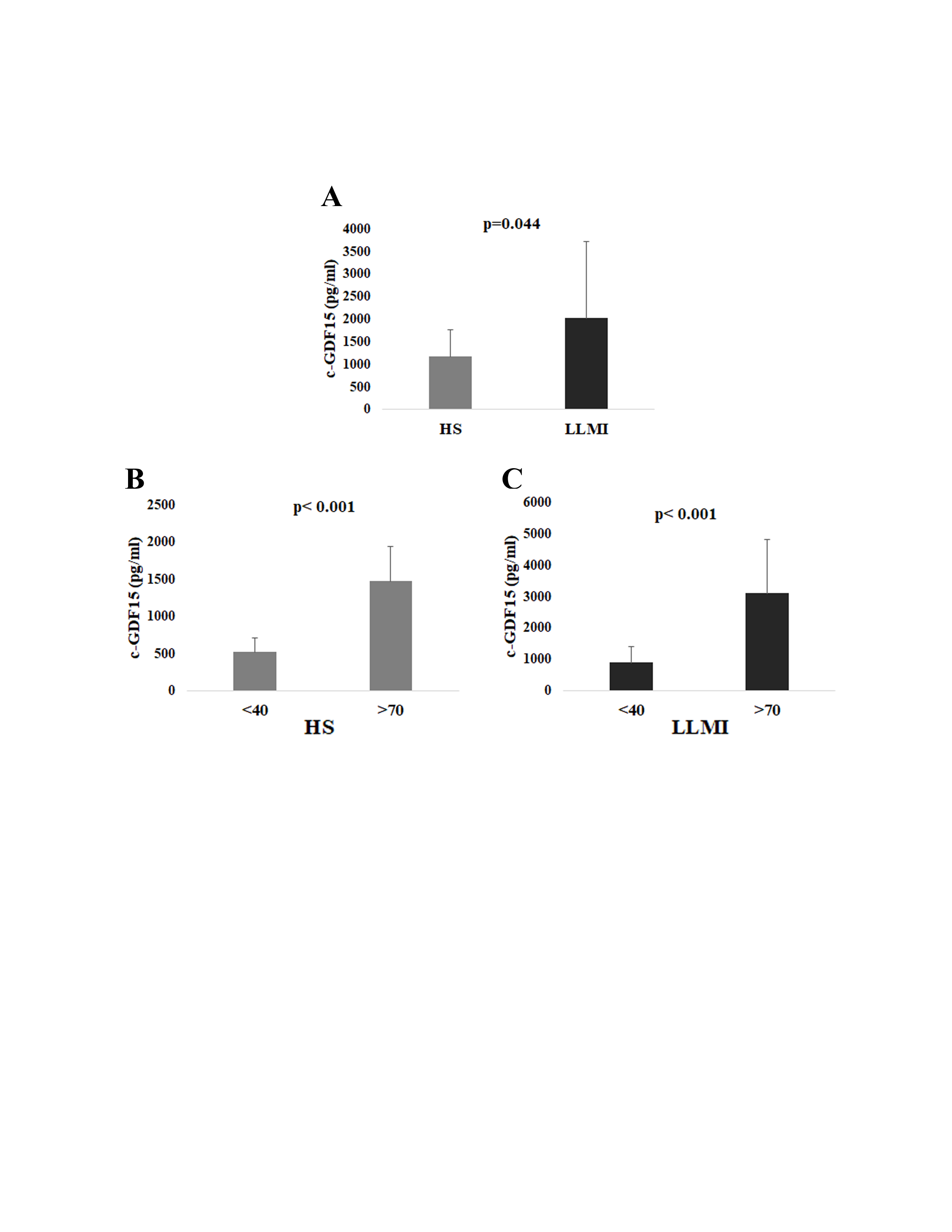

Supplement: Supplementary Figure 1 — ELISA analysis of c-GDF15 levels in healthy subjects (HS) and patients with lower limb mobility impairment (LLMI). (A) c-GDF15 level in LLMI compared to HS. (B) c-GDF15 level in HS of <40 years of age (<40) compared to HS of >70 years of age (>70). (C) c-GDF15 level in LLMI <40 compared to LLMI >70. Data are expressed as mean ± SD. Mann-Whitney test was applied. Each sample was analyzed in duplicate. [file Image_1.tif]

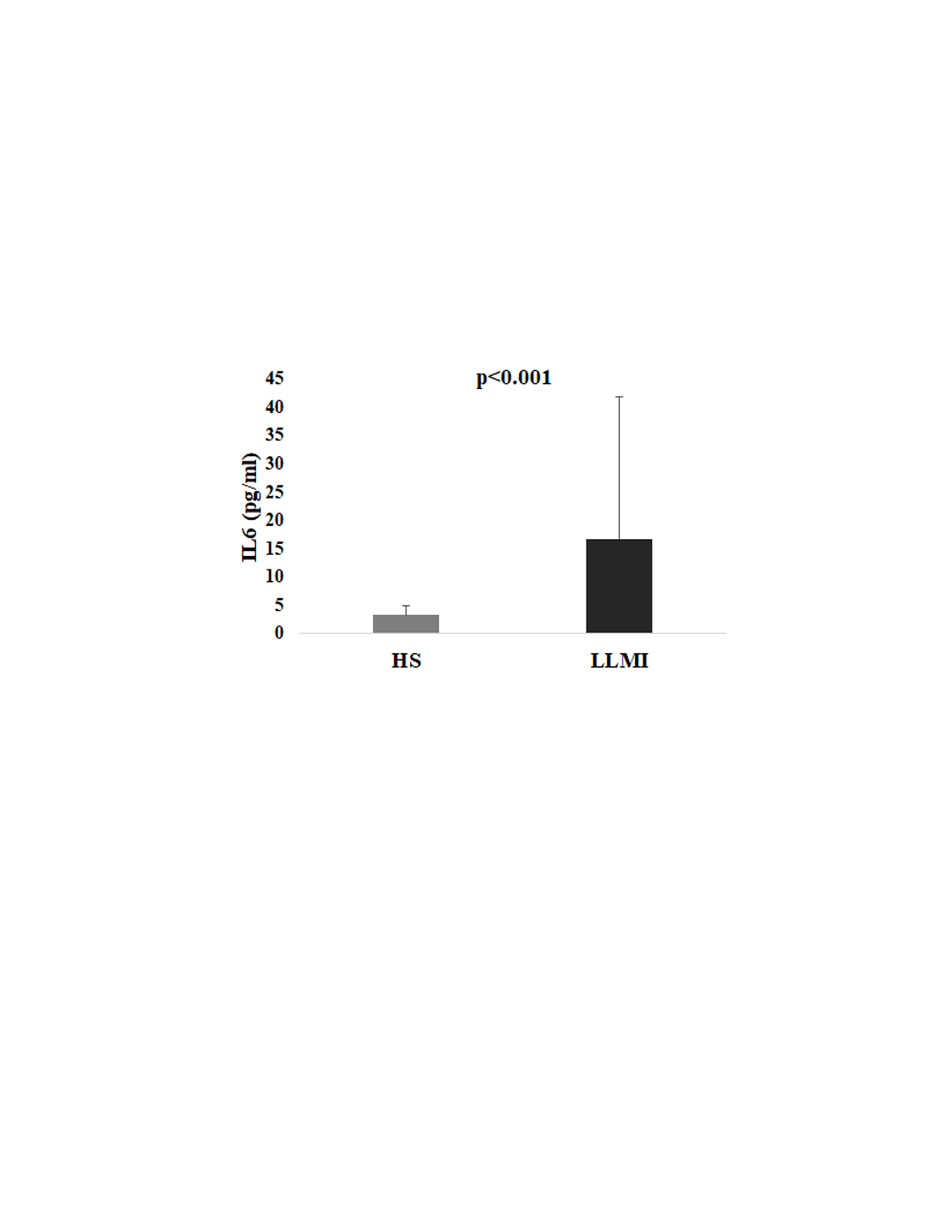

Supplement: Supplementary Figure 2 — ELISA analysis of plasma IL6 levels in HS and LLMI. Data are expressed as mean ± SD. Student’s t test was applied. Each sample was analyzed in duplicate. [file Image_2.tif]
